# Association between adaptive immunity and neutrophil dynamics in zebrafish (*Danio rerio*) infected by a parasitic ciliate

**DOI:** 10.1371/journal.pone.0203297

**Published:** 2018-09-11

**Authors:** Louise von Gersdorff Jørgensen, Rozalia Korbut, Sandra Jeberg, Per Walter Kania, Kurt Buchmann

**Affiliations:** Section of Parasitology and Aquatic Pathobiology, Department of Veterinary and Animal Sciences, Faculty of Health and Medical Sciences, University of Copenhagen, Frederiksberg C, Denmark; INRA, FRANCE

## Abstract

The protective immune response in zebrafish (*Danio rerio*) against the parasitic ciliate *Ichthyophthirius multifiliis*, targeting host skin, fins and gills, comprises an accelerated and manifold elevated immunoglobulin gene expression as well as a significantly elevated number of neutrophils at infected sites. Experimental fish were subjected to a primary *I*. *multifiliis* infection followed by a series of secondary exposures before they were challenged by a high dosage of infective theronts. Immunized fish responded immediately with a protective response suggesting existence of immunological memory whereas fish exposed to the parasite for the first time obtained a marked infection. The primary response to infection was dominated by expression of genes encoding acute phase reactants and inflammatory cytokines as well as recruitment of neutrophils at infected locations. Immunized fish showed a significantly upregulated immunoglobulin gene expression following challenge, which indicates existence of a secondary response effected by antibodies. Both responses induced a significantly elevated expression of the Th2 signature cytokine Il13. The increased presence of neutrophils in immunized fish suggests that innate cell mediated immunity supplements or influence the protective response against the parasite.

## Background

Teleost fishes are equipped with well-developed immune systems comprising both innate [1, 2] and adaptive responses [3], which is the basis for existence of successful vaccination programs in the aquaculture sector [4–6]. Although a protective immune response can be induced in fish by various forms of immunization it is still inadequately described to what extent teleosts mount a secondary immune response following re-stimulation. A classical secondary immune response as described in mammals is characterized by immunological memory and an accelerated and manifold rise of specific antibodies with a partial switch of Ig class [7]. Fish do not display a class switch [8] but may exhibit a re-inforced antibody production although relatively few studies have elucidated this process. Teleosts rely to a large extent on innate immune defenses [9–11] and neutrophils are considered a central cell type in this first line response [7] but recent evidence suggests that they also play more sophisticated roles, as they have immunomodulatory capabilities, contribute to coordination of subsequent adaptive mechanisms and conserve host integrity [12, 13]. The present study elucidates the dynamics of neutrophils and adaptive immunity. We selected a host-parasite model comprising zebrafish and the ciliate *Ichthyophthirius multifiliis* as a suitable system to investigate immune gene regulation and neutrophil behavior during an adaptive immune response.

The parasite invades the epithelia of skin, fins and gills and settles above the basal lamina in interstitial tissue spaces. The feeding stage termed the trophont feeds on active cells, cell debris and fluids in the epidermis [14, 15] and after a temperature-dependent growth period the parasite leaves the fish. Subsequent stages include the free-swimming tomont, which transforms into the tomocyst and give rise to numerous infective theronts. The duration of the trophont stage in the fish surface layers is sufficient for an induction of a protective response in several species of fish such as common carp (*Cyprinus carpio*), channel catfish (*Ictalurus punctatus*), rainbow trout (*Oncorhynchus mykiss*) and zebrafish (*Danio rerio*) [16–20], if these fish survive a first infection. The theront is able to invade both naïve and immune fish but within a few hours the majority of parasites are either killed or forced to prematurely exit the immune fish, whereas they remain in naïve fish. It has been reported that protection is associated with production of both systemic and mucosal antibodies [19–22], which bind to parasites *in situ* and *in vitro* [19, 20]. Other humoral immune factors, such as complement, have been hypothesized to play a role in the primary response against *I*. *multifiliis* since the gene encoding component C3 is regulated in infected carp [23] and rainbow trout [24, 25]. Cellular immune factors such as non-specific cytotoxic cells (NCCs), eosinophilic granular cells, basophils, neutrophils, CD8^+^ lymphocytes and MHC II^+^ cells have been detected in association with the parasite in infected fish hosts [17, 25, 26]. When elucidating the association between adaptive immunity and neutrophil dynamics we apply a high-resolution qPCR methodology and real-time imaging analyses to address the relative role of these immune factors in infected areas of naïve and immunized zebrafish following exposure and re-exposure to the parasite.

## Materials and methods

### Ethics statement

All experiments were approved under the license no. 2016-15-0201-00902 issued by the Animal Experiments Inspectorate under the Danish Ministry of Environment and Food and followed associated ethical guidelines. Fish were euthanized before severe pathology occurred.

### Fish

A total of 142 zebrafish were used for the experiments. Zebrafish were reared in a recirculated system (Aquaschwarz) at 27°C with a pH of 7.4 and conductivity of 550 μS. Ten percent water was exchanged every day and the fish were fed live *Artemia* and pelleted dry feed (ZM Fish Food) 1–3 times per day. Own cross-breeds of adult zebrafish of 5 + months (AB wildtype, semi-transparent (Tra, lacking iridophores) [27] and transparent (Tra;nac, lacking iridophores and melanophores)) [28, 29] were used for the qPCR study. A previous study determined that these variants were equally susceptible to *I*. *multifiliis* [16]. Tg(MPO:GFP)^i114^ [30] adult zebrafish were used for the *in vivo* imaging of neutrophils. All fish were acclimatized at room temperature (23°C) before initiation of the experiment.

### Immunization procedure

**Fig 1:**
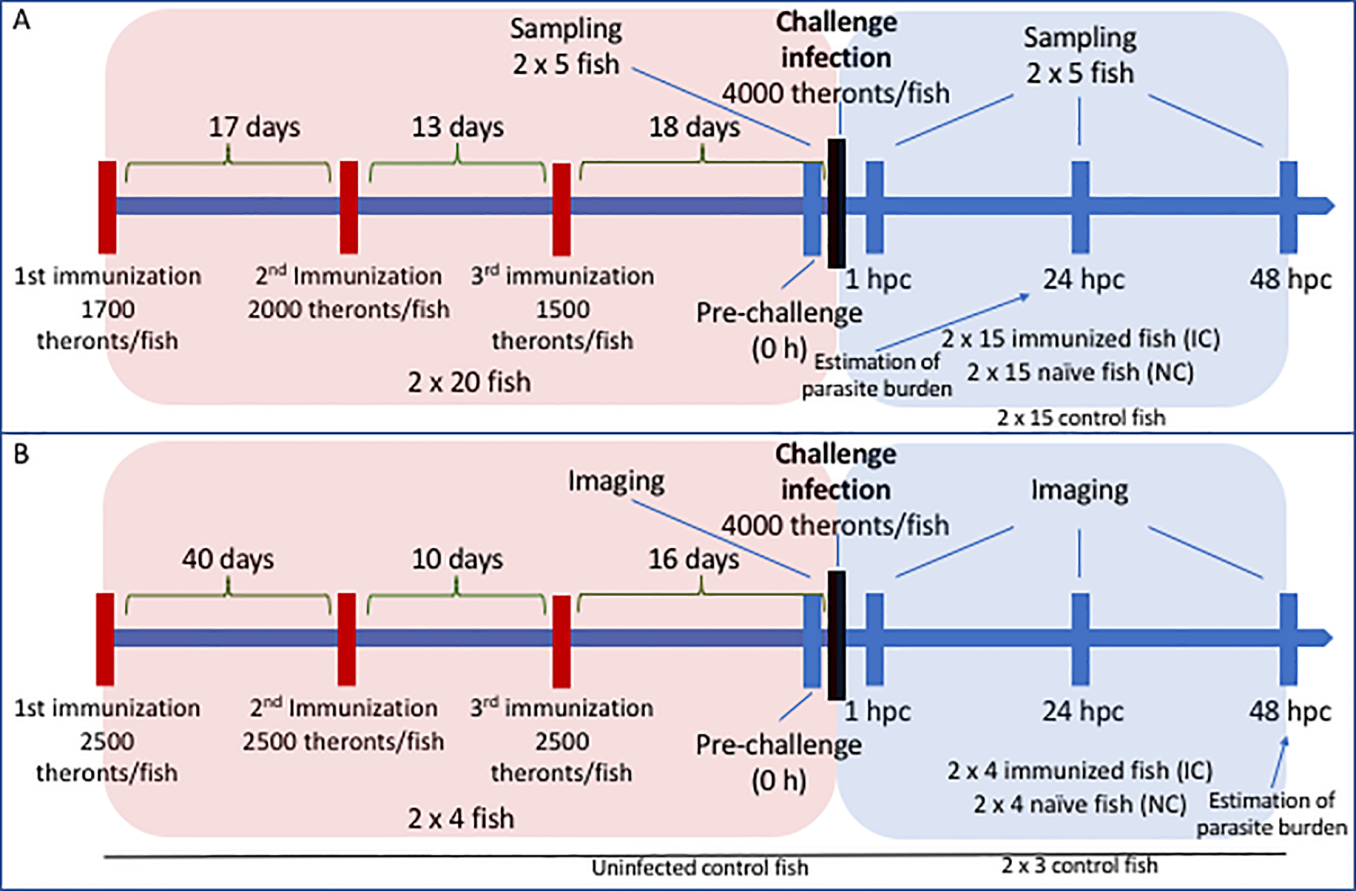
Timeline of the immunizations and challenge infections of zebrafish with the parasite *Ichthyophthirius multifiliis*. Timeline of exposures and samplings regarding zebrafish used for A) gene expression analyses and B) image analyses investigating neutrophil recruitment. The pink and the blue areas frame the periods of immunizations and challenge infections, respectively. Periods between immunizations and sampling points are indicated as well as immunization and challenge dosages of the parasite.

Parasites for the immunization and challenge procedures were produced based on a strain isolated from infected fish purchased from an ornamental fish shop. The parasite species verification was conducted morphologically identifying the ciliated parasites with the unique horseshoe-shaped macronucleus. The infected fish were euthanized in an overdose of etomidate (Sigma-Aldrich E2503000) and left for 4 h in clean fish facility water at room temperature, which allowed trophonts to exit the fish and transform into tomonts. The euthanized fish were then removed and tomonts left overnight for further development into tomocysts with subsequent release of infective theronts. The next day released theronts were counted using a dissection microscope whereafter fish were exposed to infection (1000–2500 theronts per fish) three times before the final challenge procedure (Fig 1). Fish were kept at a density of 10 fish/L for three days following infections and subsequently kept at a density of 5 fish/L. The chronology of immunizations for the fish used for qPCR and the fish used for imaging are depicted in Fig 1. Twenty-four hours post challenge (hpc) fish received a complete water change. From here on the fish were fed with dry feed and received a 50% water change every day.

### Challenge procedure

Following the immunization process described above the challenge procedure was performed comprising 2 x 15 naïve and 2 x 15 immunized fish (the qPCR study) and 2 x 4 naïve and 2 x 4 immunized fish (neutrophil analyses). In both cases an infection dosage of 4000 theronts per fish and a density of 10 fish/L were applied. This dosage normally induces high mortality in naïve zebrafish.

### Parasite burden

The total number of parasites was counted 24 h post challenge on all fins of three fish per group (qPCR study) using a stereo microscope (Leica, Germany) (magnification 7–40 x) and on the caudal fin 48 h post challenge (transgenic fish used for neutrophil counting).

### qPCR

Samples for qPCR were taken 1 hpc, 24 hpc and 48 hpc from exposed fish and non-exposed controls. Five fish per tank (10 fish per treatment) were euthanized in an overdose of etomidate (30 mg/L) at the same time and transferred to RNAlater (Sigma-Aldrich, cat.no. R0901). The gills and fins were excised from the fish while still in RNAlater and transferred to individual vials with RNAlater. Samples were kept at 4°C for 24 h, whereafter they were transferred to -20°C until further use.

#### RNA purification

RNA purification was conducted using the GenElute™ Mammalian Total RNA Miniprep kit (Sigma-Aldrich, cat. no. RTN350). In brief: Gill and fin tissues were incubated in a lysis buffer containing beta-mercaptoethanol and subsequently sonicated on ice (Sonicator Ultrasonic Liquid Processor Model XL 2020, Heat Systems). Purified RNA was DNase-treated (Fermentas, cat.no. EN052), quantified (Nanodrop 2000 (Saveen & Werner APS)) and quality assessed on a 2% agarose gel containing ethidium bromide.

#### cDNA synthesis

cDNA was synthesized using TaqMan^®^ Reverse Transcription Reaction (Thermo Fisher Scientific, cat.no. N8080234) in a T3 Thermocycler (Biometra).

#### Real-time PCR

Real-time PCR was run in an Agilent Technologies AriaMX Real-Time PCR system (AH Diagnostics) using a 3 min denaturation step at 95°C, 40 cycles of 5 seconds at 95°C followed by a combined step of annealing and elongation for 10 seconds at 60°C with Brilliant III Ultra-fast qPCR Master Mix (Agilent Technologies, cat. no. 600880). Primers and probes (S1 Table) were designed as previously described [31]. Negative controls included RT minus and mastermix plus water.

### *In vivo* imaging

Transgenic adult Tg(MPO:GFP)^i114^ zebrafish were anaesthetized one by one in etomidate in a concentration of 3 mg/L at time points pre-infection, 1 hpc, 24 hpc and 48 hpc and when the fish were completely immobilized images of the caudal fin were obtained using normal light and GFP filters (488 nm) with a fluorescence stereo microscope (Leica MZ FLII). Only the fins of adult fish are adequately thin and transparent to distinguish single cells. Using ImageJ the dorsal posterior quadrant of the caudal fin from the center of the fork was marked up according to [14] and all neutrophils within this area were counted manually. Manual counting was chosen because it gave a more accurate result as it allowed us to disregard autofluorescence in pigment spots on the tail fin by comparing the image obtained using GFP filters with the image using normal light (identifying the pigmentation spots). Cell densities are presented in cells/mm^2^.

### Data analyses

Parasite infection levels in challenged fish (naïve and immunized) were compared and analyzed using a Mann-Whitney statistical test (P<0.05). The 2^-ΔΔCt^ method by Livak & Schmittgen (2001) [32] was used for relative gene expression determination. An average of three reference genes were used for normalization: *eukaryotic translation elongation factor 1 alpha 1*, *like 1 (eef1a1l1)*, β*-actin* and *ribosomal protein L13a* (*rpl13a)*. Data were compared for both treatments at each time point in relation to non-infected controls and immunized fish were furthermore compared to fish receiving a first infection using Student’s t-test. A P value of <0.05 and more than 2 fold regulations were considered significant. Some genes were expressed at a low level, especially in non-exposed fish, and the qualitative regulations were assessed by a Mann-Whitney *U*-test at the same probability level. In the main text fold changes are provided in whole numbers but in the S1 Appendix fold changes are given with two decimals.

Duplicate groups were collapsed into single groups since no significant difference was observed with regard to neutrophil numbers (Student’s t-test, P<0.05). Controls, fish infected for the first time and infected immunized fish were tested against each other with a one way ANOVA using Tukey's multiple comparisons test and a P value of <0.05. All materials and data are available to readers.

## Results

Fish receiving only one *I*. *multifiliis* exposure are designated NC (Naïve Challenged) and the fish exposed several times before challenge are designated IC (Immunized Challenged). Non-exposed control fish are referred to as controls.

### Parasite burden

**Fig 2:**
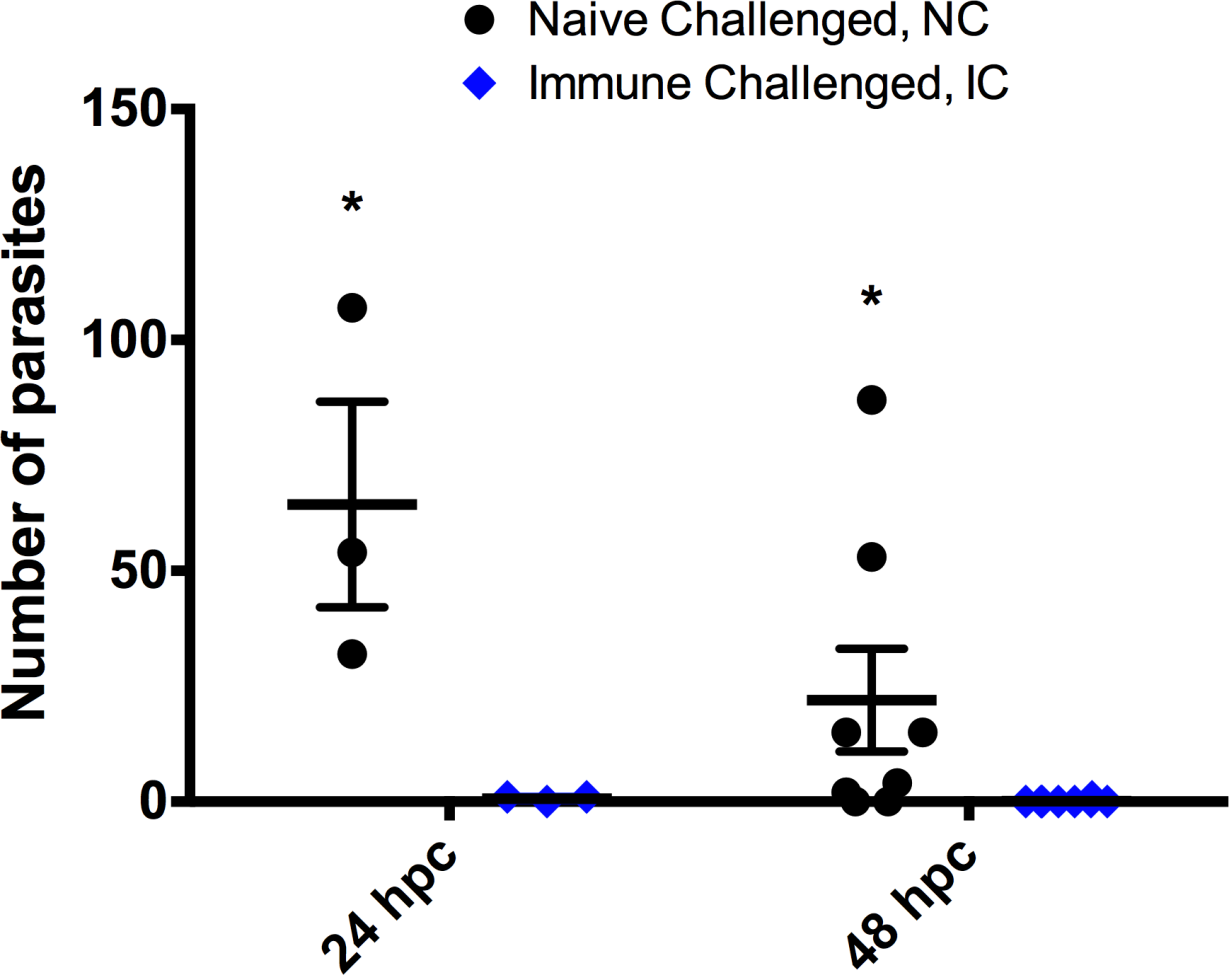
Parasite burden of zebrafish (*Danio rerio*) infected with the parasite *Ichthyophthirius multifiliis*. The parasite burden in fins of naïve challenged (NC) and immune challenged (IC) zebrafish with *I*. *multifiliis*. Significant difference (p<0.05) is indicated with *.

To estimate the level of protection and susceptibility of zebrafish receiving a challenge with *I*. *multifiliis*, the parasite burden was determined. Parasites were counted on the fish used for qPCR at 24 hpc and on the transgenic fish used for imaging at 48 hpc (Fig 2). Naïve challenged fish harboured an average of 64.3 (STD 38.6) parasites per fish on the fins (all fins) and immune challenged fish carried one or less parasites per fish 24 hpc. One day later NC fish harboured an average of 22 (STD 31.6) on the caudal fin (only fin included in the imaging study) and IC fish carried 0.1 (STD 0.4) parasites per caudal fin. Both results were statistically significant using a Mann-Whitney test.

### qPCR

**Fig 3:**
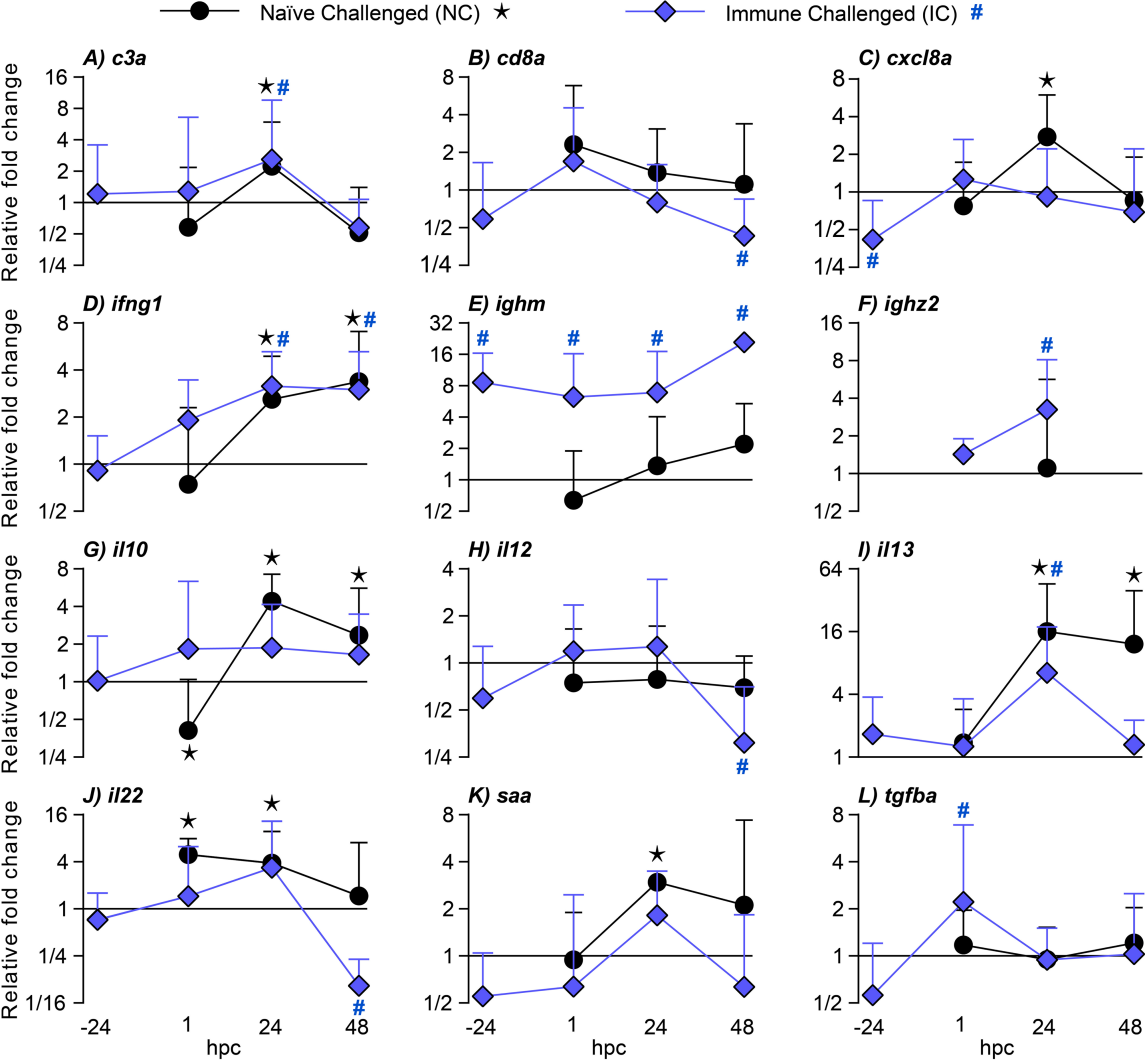
Gene expressions in *Ichthyophthirius multifiliis* infected fins (primary and secondary infections) of zebrafish (*Danio rerio*). Regulations are shown before challenge and 1 h, 24 h and 48 h post challenge. The standard error of mean (SEM) is indicated as error bars and 12 out of 26 investigated genes are shown in the figure. Significant differences (p<0.05) compared to uninfected controls are indicated with a star for naïve challenged (NC) fish and a hashtag for immune challenged (IC) fish.

**Fig 4:**
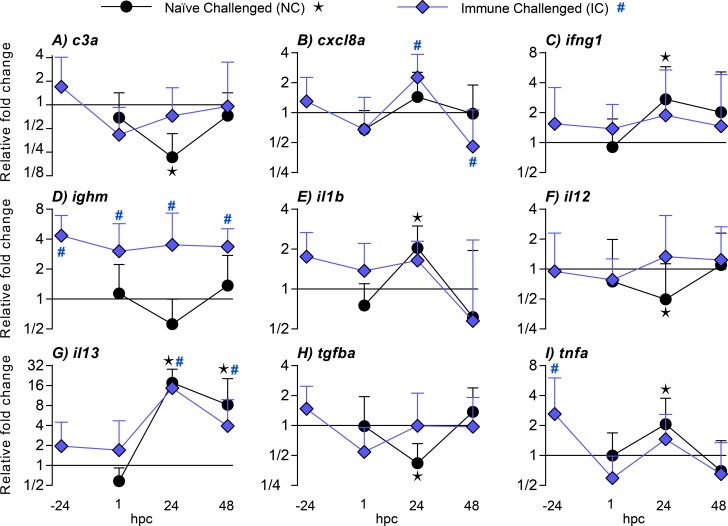
Gene expressions in *Ichthyophthirius multifiliis* infected gills (primary and secondary infections) of zebrafish (*Danio rerio*). Regulations are shown before challenge and 1 h, 24 h and 48 h post challenge. The standard error of mean (SEM) is indicated as error bars and 9 out of 26 investigated genes are shown in the figure. Significant differences (p<0.05) compared to uninfected controls are indicated with a star for naïve challenged (NC) fish and a hashtag for immune challenged (IC) fish.

For investigation of primary and secondary immune responses to *I*. *multifiliis* a gene expression analysis was performed. A total of 26 immune genes were investigated but only significantly regulated genes are described in detail in the result section (Figs 3 and 4). A complete set of gene expression data are presented in the S1 Appendix.

### Gene expression analyses

#### Fins

We investigated a range of cytokines to elucidate the gene expression profile of the primary and the protective response in relation to the Th pathways, which are known in mammals. The Th1-associated cytokines investigated comprised *interleukin 12a* (*il12a*) and *interferon gamma 1* (*ifng1*). Two days after challenge *il12a* was down-regulated in IC fish and *ifng1* was up-regulated (3 fold) in both NC and IC fish one and two days after challenge.

The expression of the cytokine gene *interleukin 13* (*il13 (il4/13a))*, which is associated with the Th2 pathway in mammals, increased with 12–16 fold in NC fish. The gene was less up-regulated in IC fish (6 fold) at 24 hpc and returned to an un-regulated state at 48 hpc (Fig 3 and S1 Appendix).

Cytokine genes related to the Th17 pathway in mammals comprised *interleukin 6* (*il6*), *22* (*il22*) and *23*, *alpha subunit p19* (*il23a*). Il6 has many functions and the regulation of this gene is described in the miscellaneous paragraph below. Whereas *il22* was up-regulated in NC fish (4–5 fold) it was down-regulated 9 fold in NC fish 48 hpc. Using the qualitative approach *il23a* was significantly down-regulated in IC fish relative to controls.

Investigations of the mammalian regulatory T cell (Treg)-associated pathway included the cytokines *transforming growth factor*, *beta 1a* (*tgfb1a)* together with the transcriptional factor *forkhead box p3a (fox p3a)*, which controls the differentiation and function of regulatory T cells. An un-regulated state for *fox p3a* was found whereas *tgfb1a* was up-regulated 2 fold in IC fish immediately following challenge (1 hpc).

A range of genes not specifically associated to mammalian Th pathways were also studied (referred to as miscellaneous in the S1 Appendix). The pro-inflammatory cytokines *interleukin 1*, *beta* (*il1b)*, *il6*, *tumor necrosis factor a (TNF superfamily*, *member 2)* (*tnf*a) as well as the anti-inflammatory cytokine *interleukin 10* (*il10)* were investigated. A significant elevation, assessed by the qualitative analysis, of *il6* in immune challenged fish was observed one day after challenge compared to uninfected controls. The *il10*-gene was only regulated in naïve challenged fish with a down-regulation immediately after challenge followed by an up-regulation (4 fold, 24 hpc), which decreased almost to normal expression at 48 hpc. *Chemokine (C-X-C motif) ligand 8a* (*cxcl8a*), is produced by many cells including macrophages and may act as a chemokine with the ability to attract functional cells (such as neutrophils) to infection areas but only NC fish responded with an elevation of *cxcl8a* (3 fold) at 24 hpc.

Regulation of genes associated with cellular markers were investigated for B cells (*immunoglobulin heavy constant mu* (*ighm)*, *immunoglobulin heavy constant zeta* (*ighz*) and *immunoglobulin heavy constant zeta2* (*ighz2*), macrophages (*macrophage expressed 1*, *tandem duplicate 1* and *2 (mpeg1*.*1* and *mpeg1*.*2*) and T cells (*CD4-2 molecule*, *tandem duplicate 1 and 2* (*cd4-2)* and *CD8a molecule* (*cd8a*)). The gene *ighm* was up-regulated in naïve challenged fish (2 fold) only at 48 hp, whereas an up-regulation was observed throughout all time points in immune challenged fish (6, 7 and 21 fold at 1 h, 24 h and 48 hpc, respectively). The gene encoding the mucosal antibody Ighz2 was also up-regulated in IC fish at 24 hpc (3 fold) however *ighz2* was insufficiently expressed to conduct analyses at 1 and 48 dpc. NC fish displayed a slight up-regulation of *cd8a* immediately following challenge while a down-regulation was observed (2 fold) in IC fish two days after challenge.

We followed other relevant immune genes encoding *toll-like receptors* 2 and 3 (*tlr2* and *tlr3)*, the acute phase protein *serum amyloid A* (*saa*) and *complement component c3a*, *duplicate 1*, *2* and *3* (*c3a*). The gene *saa* was only up-regulated in NC fish (3 fold) while *c3* was up-regulated 2–3 fold in both NC and IC fish. The I-antigen52B, which is a surface protein of *I*. *multifiliis* was expressed (present or not present) only when the parasite occurred in the samples as judged from the microscopic analysis. In un-infected controls expression was 0, whereas 90%, 60% and 50% of the fin samples in NC fish at 1 h, 24 h and 48 hpc, respectively expressed the *I-antigen52B*. In IC fish 60%, 20% and 0% at 1 h, 24 h and 48 hpc, respectively expressed the gene.

#### Gills

The genes investigated using fins were also investigated in gills tissue (Fig 4 and S1 Appendix). The cytokine gene *il12a* was slightly down-regulated (2 fold, 24 hpc) in NC fish while *ifng1* at the same time point was up-regulated (3 fold).

The gene encoding the Th2-associated cytokine *Il13* was highly up-regulated in NC fish (18 fold, 24 hpc) and in IC fish (15 fold, 24 hpc).

Using the qualitative assessment for the Th17-associated gene *il23a* an elevated expression was observed one day after challenge in NC fish, while *tgfb1a* at the same time point was down-regulated.

Transcription of the pro-inflammatory cytokines Il1b and Tnfa was up-regulated in NC fish one day after challenge. Tnfa is, besides it’s inflammatory capabilities, associated with both Th1 and Th2 pathways in mammals. Immune challenged fish showed an up-regulation of *cxcl8a* one day after challenge (2 fold), which was followed by a down-regulation two days after challenge (2 fold).

As in fins, immune challenged fish had a higher level of transcripts of the antibody Ighm in the gills (3, 4 and 4 fold at 1, 24 and 48 hpc, respectively) whereas no regulation was observed in naïve challenged fish. A qualitative analysis of the *ighz2*-immunoglobulin gene revealed a slight increase in the immune challenged fish compared to naïve challenged fish immediately following challenge (1 hpc).

The gene encoding the complement factor C3a was down-regulated in naïve challenged fish (5 fold, 24 hpc) and up-regulated in immune challenged fish (2 fold, 1 hpc). The expression pattern of the gene for the parasite surface protein I-antigen52B was similar to the pattern observed in fins with an approximate equal parasite burden immediately after challenge (1 hpc) between NC and IC fish. This was followed by a reduction in the parasite burden in the susceptible fish (NC) and a drop to zero in protected fish (IC).

### Imaging

**Fig 5:**
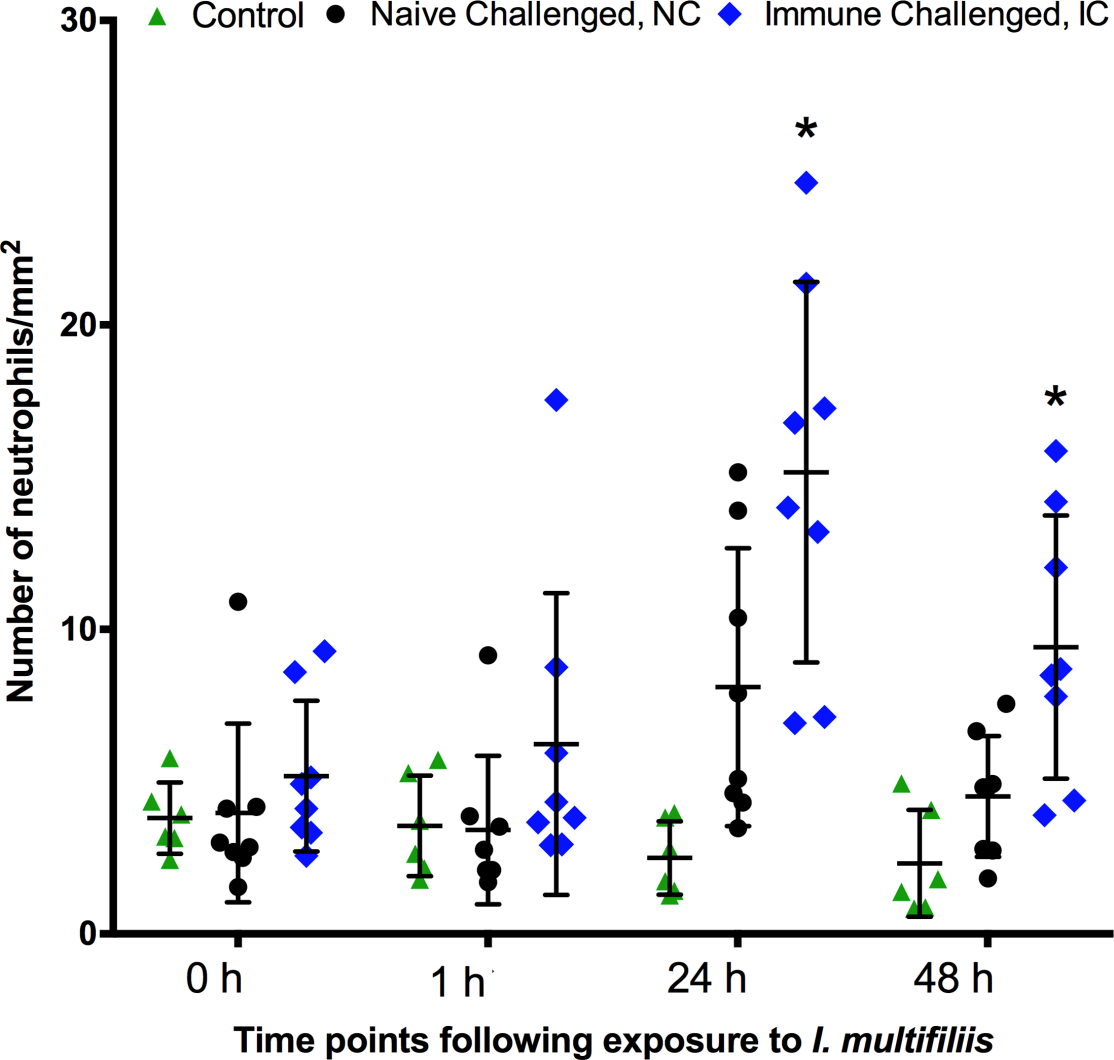
Neutrophil numbers in *Ichthyophthirius multifiliis* infected fins (primary and secondary infections) of zebrafish (*Danio rerio*). Groups comprise uninfected control fish, naïve fish infected for the first time (NC) and immunized fish receiving a challenge exposure (IC). * represents statistically significant difference (P<0.05) from the control and the naïve NC fish.

**Fig 6:**
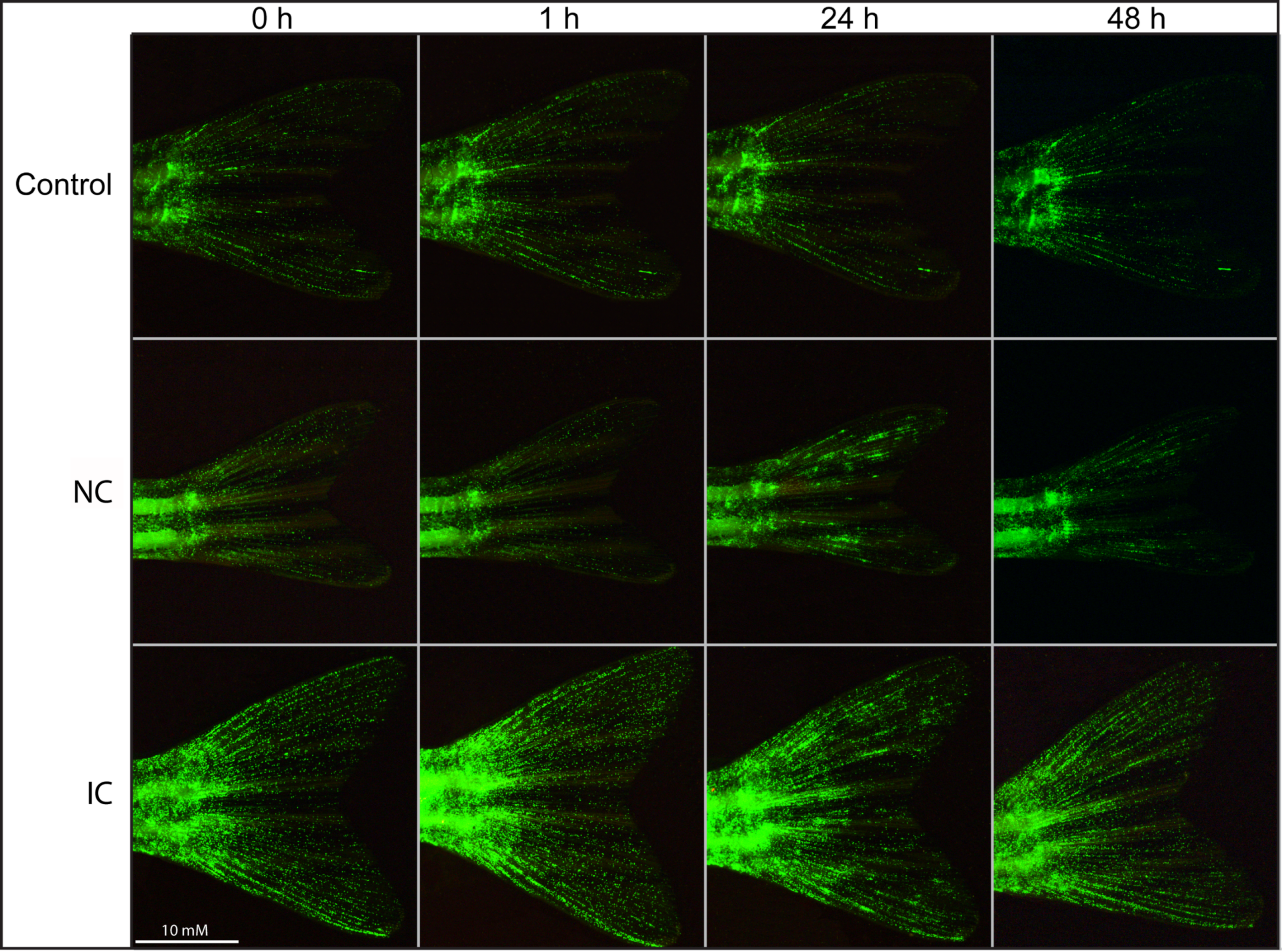
GFP-labeled neutrophils of *Ichthyophthirius multifiliis* infected zebrafish caudal fins (primary and secondary infections). Representative images of the caudal fins demonstrating the recruitment of neutrophils to infected areas following exposure to the fish parasite *I*. *multifiliis* of naïve (NC) and immunized (IC) adult zebrafish. The scale bar applies to all images.

To analyze the association between neutrophil dynamics and a protective immune response against *I*. *multifiliis* the neutrophil recruitment during a primary and a secondary immune response was investigated. The number of neutrophils in the caudal fins of immune challenged fish was significantly higher (15.2 cells/mm^2^ (STD 6.3), 24 hpc) compared to naïve challenged fish (8.1 cells/mm^2^ (STD 4.6), 24 hpc) both one and two days post exposure to the parasite (Fig 5). Images of the caudal fin revealed that in both NC and IC fish focal accumulation of neutrophils occurred (Fig 6) one day after challenge. A closeup of neutrophils on the fin from the IC fish in Fig 6 and a closeup of a parasite on the fin as well as an image of a wildtype tail fin obtained with GFP filter settings is presented in the S1 Fig.

## Discussion

Immunological memory is crucial for survival of fish and higher vertebrates and is a prerequisite for raising a protective response and obtain a successful vaccination result [4–6]. The secondary immune response in mammals is characterized by an accelerated, rapid and increased specific antibody production, which is associated with isotype switching and affinity maturation [33–35]. In this study we demonstrated that zebrafish develop a protective response against re-infection with *I*. *multifiliis*. This was shown as an accelerated and fast clearance of *I*. *multifiliis* from exposed host tissue as judged by the microscopic analysis and a drastic decline of parasite gene transcripts, which corresponded to a previously described clearance of parasites from protected fish [16, 17, 36]. Molecular methods for measuring a series of immune reactions, including antibody production, are available for zebrafish [31] and in the present study the occurrence of a secondary immune response was shown in zebrafish by the highly increased expression levels of genes mainly associated with antibody production. It is noteworthy that expression of the immunoglobulin genes *ighm* and *ighz2* together with the Th2 signature cytokine gene *il13* was highly elevated following re-infection. In several fish species indications of a differentiated Th1 and Th2 response, as defined in mammals have been described [37–39] but knowledge is scarce in this field. During evolution, fish were the first vertebrates to develop adaptive immune responses but both bony and cartilaginous fish rely to a great extent on robust innate defenses, which may involve acute phase reactants and inflammation. This was confirmed in the present study showing upregulation of several cytokines during the first infection. The cellular arm of the innate response involves neutrophils, which are highly motile phagocytes playing critical roles in protection towards damage or infection. It has been suggested that this cell type has a series of functions in both innate and adaptive immunity [12, 13] and the present study has demonstrated elevated occurrence of neutrophils during elimination of parasites concurrent with highly increased expression of Ig and Th2-associated cytokine genes. The association may not be causal but it cannot be excluded that the adaptive response is influenced by these cells or vice versa. We recorded by the use of bio-imaging techniques that neutrophils were recruited to the infection foci in the caudal fin one day after challenge in both NC and IC fish but the latter immunized group exhibited a significantly higher number of neutrophils in the infected area. Invading parasites (theronts) and the established parasites (young trophonts) almost disappeared from protected fish fins within 24 h post-challenge and the increased density of neutrophils may have played a role in this early protective response. A *Gyrodactylus derjavini* infection was previously found to induce moderate protection against a subsequent *I*. *multifiliis* infection confirming that non-specific responses are protective [40]. It was recently reported that neutrophils in mammals are able to entrain macrophage polarization to battle parasites using release of Il13 [41] and that a sub-set of neutrophils are capable of inducing T-cell independent antibody production [42]. Here, the fluctuations in neutrophil numbers correlate well with the production of *il13* transcripts and slightly delayed *ighm* transcripts (Figs 3 and 4), which suggests that teleostean and mammalian neutrophils may have analogous functions.

The gene expression analyses targeting the gene encoding *I*. *multifiliis* surface protein I-antigen52B showed that naïve and immune challenged fish became equally infected immediately following challenge (1 hpc) in fins and gills but that the immunized fish eliminated the infection rapidly. At the last two sample points IC fish had almost eliminated the parasites, while NC fish merely managed to moderate the infection. *I*. *multifiliis* parasites increase in size for at least three to five days in zebrafish [16] and it has been shown by *in vivo* imaging that the parasites evade the immune system by ingesting active immune cells [14]. Within a few days the parasites grow from a diameter of 30 μm up to 1 mm (size increase 37,000 x), which is based on ingestion of host tissue and it may be speculated that the massive ingestion of cells by the parasites may at least partly explain the decreased immune signaling.

Immunological memory in fish has been described previously [43, 44] and Rijkers *et al*. (1980) [45] showed that repeated injection of antigens into common carp elicited a secondary immune response, as reflected by an accelerated and highly increased appearance of antibody producing cells. In contrast, corresponding knowledge on immunological memory induced by antigen exposure of fish surfaces is limited. Repeated immersion vaccination of rainbow trout against the bacterial pathogen *Yersinia ruckeri* increased protection but the cellular and humoral parameters were not manifold increased. It was suggested that the lack of a classical secondary immune response in that system (30 sec immersion of fish into an *Y*. *ruckeri* antigen solution) was caused by the limited antigen amount passing into the host [46]. Infection with *I*. *multifilii*s causes an extended stimulation time as the trophonts remain feeding in the epidermis for several days before exiting. Besides activation of immunoglobulin genes following re-exposure to *I*. *multifiliis* a signature Th2 cytokine was upregulated concomitant with downregulation of Th1 and Th17 genes. This suggests that a Th1/Th2-like switch takes place during the immune response against the parasite, which also has been reported previously in rainbow trout infected by the ecto-parasite *Ichthyobodo necator* [47].

Following the first parasite exposure we recorded a primary immune reaction in the fins, which basically comprise two epidermal layers, capillaries, blood and fin rays. It involved regulations of a variety of genes encoding cytokines associated with the mammalian Th1 (*il12a*, *ifng1*), Th2 (*il13*) and Th17 (*il22*, *il23a*) responses, reflecting an inflammatory response. The secondary immune response in IC fish had a different profile as mainly a Th2 associated cytokine gene (*il13*) was highly upregulated whereas Th1 (*il12a*) and Th17 (*il22*, *il23a*) cytokine genes were downregulated. The only exception was the cytokine *ifng1*, which also was slightly up-regulated in protected fish. Il12 is involved in differentiation of naive T cells into Th1 cells in mammals [48] and interferon gamma is a pro-inflammatory cytokine with regulatory functions in both innate and adaptive immunity (including activation of macrophages and augmentation of antigen presentation) [49]. Il4 is a mediator of Th2 adaptive immune responses in mammals and induces B cell proliferation and antibody production. The zebrafish equivalent to mammalian Il4 is likely Il13 [50] and recombinant Il13 augments the density of IgM^+^ B and IgZ-2^+^ B cells in peripheral blood [50, 51]. The high up-regulation of *il13* transcripts in fins and gills may therefore indicate that Il13 has a prominent role in the response against *I*. *multifiliis*.

Regulations of the Th17 associated genes were merely seen immediately after parasite exposure of naïve challenged fish. Il22, an activator of antimicrobial peptide genes in macrophages [49, 52], targets Il22 receptors at mucosal-associated tissues [53], such as fish skin and gills. The up-regulated state of *il22* lasted 24 h, whereafter it was downregulated in NC fish framing that this event merely reflects an inflammatory reaction. In IC fish a significant down-regulation (9 fold) was observed 48 hpc in fins, which further support the notion of Th17 inhibition in protected fish.

Tgfb is a cytokine secreted by macrophages [49, 54] and is primarily associated with repair functions and with Treg cell generation in mammals. It may affect Th17 differentiation [54], inhibit T and B cell proliferation and stimulate macrophage and NK cell activity in zebrafish [55]. The *tgfb1a* up-regulation observed in fins of protected fish one day after challenge and the down-regulation in gills at the same time point in NC fish may indicate that macrophages and repair processes are stimulated during the first period of time after infection until a potential protective response against *I*. *multifiliis* has shown effect.

The dynamic expression of genes encoding immunoglobulin cell markers strengthen the impression of a Th2-biased response to *I*. *multifiliis* in zebrafish. IgM is the most abundant antibody in fish blood and plays a major role in immune responses due to its ability to inhibit, agglutinate and assist complement guided killing of invading pathogens. It has previously been reported that *I*. *multifiliis*-immune rainbow trout and channel catfish respond to infection with a production of antibodies, which are able to immobilize the infective stage of the parasite *in vivo* and *in vitro* [19, 20, 56]. In this study, zebrafish also responded with an elevated level of *ighm* in fins and gills of immune challenged fish at all sample points. Increased expression of *ighm* was also observed 48 h following the primary infection (NC) suggesting an early onset of an antibody mediated immune response against the parasite. The immunoglobulin subclass Ighz2 is expressed in both primary and secondary lymphoid tissues of zebrafish and is facilitated by Il4 [51]. We showed a significant up-regulation of *ighz* in fins of IC fish suggesting occurrence of an antibody mediated protective response, which complies with earlier observations in rainbow trout, where the mucosal antibody IgT (equivalent to IgZ) [57] plays a major role in protection against *I*. *multifiliis* [19, 21, 22]. Cd8 is predominantly expressed in cytotoxic T cells associated with a Th1 response in mammals. The down-regulation of *cd8a* in immunized zebrafish fins observed in this study further supports the notion that the protection is associated with a Th2-biased response.

The pro-inflammatory cytokine Il1μ, produced in macrophages [58] and neutrophils [59], is associated with cell proliferation, differentiation and apoptosis and attracts innate cells to areas of injury. The up-regulation of *il1b* in naïve challenged fish in gills reflects the inflammatory state initiated by theront invasion, corresponding to previous findings in carp skin where *il1b* and *tnfa* were upregulated following theront exposure [60]. Il6 is secreted by T cells and macrophages in mammals and has several effects targeting haematopoiesis (including neutrophil production), acute phase protein synthesis, promotion of Th2 differentiation and inhibition of Th1 polarization [61]. The upregulation of *il6* in fins of IC fish one day after challenge is in this regard in harmony with the notion that the adaptive response is Th2-biased.

Il10 is immune regulating and promotes B cell differentiation and IgM antibody secretion in an antigen specific manner in fish [49]. The down-regulation of *il10* immediately upon infection of NC fish with a subsequent elevation in fins 24 and 48 hpc may reflect that an early inflammation becomes controlled by Il10. It complies with the lack of inflammation in immune challenged fish, where the *il10* gene was un-regulated. The regulation of *il1b*, *tnfa* and *ifng1* follows the same pattern in the gills with an up-regulation during early inflammation and a return to normal levels after 48 h. Cxcl8a is a potent chemokine recruiting neutrophils to the site of injury [62] and the upregulation in fins of naïve challenged fish (24 hpc) correlates well with the concomitant influx of neutrophils observed in the tail fin. This chemokine binds to chemokine receptors Cxcr 1 and 2 and genes encoding these receptors were previously found up-regulated during an *I*. *multifiliis* infection in common carp [60]. In the gills only IC fish showed a significant up- and subsequently down-regulation of *cxcl8a* transcripts, which may be explained by the slightly higher burden of parasites compared to NC just after invasion (1 hpc) as judged by the qPCR data. This suggests that *cxcl8a* was up-regulated due to the high invasion and subsequently down-regulated once the infection was controlled (48 hpc).

Up-regulation of the acute phase gene *saa* in the fins of naïve challenged fish indicates that there is an extra-hepatic production of Saa during a first encounter with the parasite. Saa is important during the acute phase response of inflammation and has a role in the recruitment of immune cells to inflammatory areas. In a previous study Saa transcripts were also found to be up-regulated in rainbow trout during a first infection with the parasite [63] but its effector function in relation to this disease is not established. C3 is a key component of the complement system, central in innate and adaptive immunity, and known to be regulated in spleen, head kidney and skin of rainbow trout [18, 24] and in the liver of common carp [23] during *I*. *multifiliis* infections. In the present study *c3a* was up-regulated in the fins of NC and IC fish and the C3a factor may bind directly or indirectly to *I*. *multifiliis* and may thereby play a part both in the innate and the adaptive responses. In the gills however, *c3a* was found to be down-regulated in both NC and IC fish, which may indicate that immune reactions of fins and gills differ in some respects in response to parasite infections.

## Conclusion

The present study has described the dynamic association between immune gene expression and neutrophil activity during primary and secondary immune responses of zebrafish towards a parasitic ciliate. The primary response was dominated by expression of genes encoding inflammatory elements and zebrafish seem to possess innate mechanisms, which reduce the parasite burden within a few days. Although class switching does not occur in teleosts a number of classical characteristics associated with a secondary immune response could be detected in immunized fish. Immunological memory, manifold elevated expression of immunoglobulin genes and a cytokine profile supporting Ig production were prominent in protected zebrafish challenged with infective theronts. Immunized fish responded with an antibody mediated gene expression profile concomitant with occurrence of an increased density of neutrophils in affected tissues. This association may not be causal but it cannot be excluded that the protective response is influenced by these cells or vice versa.

## Supporting information

S1 Table Primers and probes used for qPCR. Gene name, primers, probes, length of the product in base pairs (bp) and GenBank accession number is provided.
(DOCX)

S1 Appendix A complete list of qPCR results and Cq values. Data obtained from fins and gills of naïve and immune challenged zebrafish (*Danio rerio*) following several immunizations with the parasite *Ichthyophthirius multifiliis*.
(XLSX)

S1 Fig Close-up of neutrophils, the parasite *Ichthyophthirius multifiliis* and an image of a Wild Type tail fin using GFP settings. A) Enlargement of a section on a fin from Fig 5 (0h, IC) to show the neutrophils in the transgenic zebrafish line Tg(MPO:GFP)i114 in greater detail. B) Neutrophil accumulation in connection with *I*. *multifiliis* on the tail fin of an adult zebrafish of the same transgenic line. C) A wild type zebrafish tail fin using GFP filter illumination. The white arrow indicates the parasite.
(DOCX)
