# Supplementary material for: Association between adaptive immunity and neutrophil dynamics in zebrafish (Danio rerio) infected by a parasitic ciliate
Source: PLoS One. 2018 Sep 11;13(9):e0203297. doi: 10.1371/journal.pone.0203297 (PMC6133357; doi:10.1371/journal.pone.0203297)
Supplement: S1 Table — Gene name, primers, probes, length of the product in base pairs (bp) and GenBank accession number is provided. (DOCX) [file pone.0203297.s001.docx]

**Association between adaptive immunity and neutrophil dynamics in zebrafish (*Danio rerio*) infected by a parasitic ciliate**

Louise von Gersdorff Jørgensen, Rozalia Korbut, Sandra Jeberg, Per Walter Kania & Kurt Buchmann

S1 Table

| **Gene name** | **Forward primer** | **Reverse primer** | **Probe** | **bp** | **GenBank Accession number** |
| --- | --- | --- | --- | --- | --- |
| *acbt2* | CCATCCTTCTTGGGTATGGA | ACAGGTCCTTACGGATGTCG | TGCGGTATCCACGAGACCACC | 91 | BC154531 |
| *c3a* | TGCTGTTCTTCTCTCCTCAGC | WGAKGAACCCACTCTCAGCA | CTCACACTGTGTGACCCGCTAT | 88 | NM_131242 NM_131243 NM_001037236 |
| *cd4-2* | CCAGACTGGAGCCAAGAGAC | CAGTGCAGCTCCACATCACT | CCTGCGCACAGTCAGGGAAA | 133 | EF601915 |
| *cd8a* | TCGGAGGTTGTGGACTTTTC | TAATGGTGGGGACATCGTCT | CGTTCTGCTGATCGCAACCA | 88 | BC114236 |
| *eelf1a1l1* | GAACGACCCACCCATGGAGG | TGATGACCTGAGCGTTGAAG | GAACGACCCACCCATGGAGG | 158 | AY422992 |
| *fox p3a* | GACTTCTGCAACCTGCATCA | CGTATCATGGAGGCGTAGGT | GAGAGAGCAGCAACTGAAGCGC | 181 | FM881778 |
| *Iag52b* | CTGCTGGCACTCTTGTTAC | CATTCAGTACATGTATCAGTACC | ACGATTGCCCTGCTGGTACA | 343 | AF405431 |
| *ighm* | TGCAGTTCTGGTTCTGATGG | TGCACAAAATCGCTCAAATC | AATCACCCTCGGCTGCTTGG | 122 | AY643753 |
| *ighz* | ATTGGATGTCTGGCCTCTGA | AATGCTGGGTGACGTTTTTC | TGCACAAAATCGCTCAAATC | 91 | AY643750 |
| *ighz2* | CCTGACCAATTTTATCCAATACCC | GGGGTAGAAATCCTCCATAACAC | AAGCAATACACCAAGAGGGCACA | 244 | EU732710 |
| *il10* | CTTGCCAAAATCCCTTTGAA | ATCAAGCTCCCCCATAGCTT | TGAAAAGATGAAGGAAAAGGGGG | 92 | BC163038 |
| *il12* | AGCAGGACTTGTTTGCTGGT | TCCACTGCGCTGAAGTTAGA | TAACTCGTCCTGCTCGGCCC | 145 | AB183001 |
| *il1b* | CGCTCCACATCTCGTACTCA | ATACGCGGTGCTGATAAACC | GAAGGAGACCGGCAGCTCCA | 166 | BC098597 |
| *il22* | GGATTACGCCAAAGGTGAAA | CGAGCACAGCAAAGCAATAA | CGACATCGAGGAACAACGGTG | 187 | NM_001020792 |
| *il23a* | ATTACCGACCTGCCAGTGAC | TTCAGGAGGTGGCCAGTAAC | AAATGCCGAACAGATCGCCA | 212 | FN869917 |
| *il13* | GTCAGGCTGAGGAGGAGATG | AGCAGCGTGACTCCTGATCT | CTGGCCTGTCCGGTGTCAAA | 155 | AB375404 |
| *il6* | AGACCGCTGCCTGTCTAAAA | CAACTTCTCCAGCGTGATGA | TCCGCATGGACTCGCAAGAC | 293 | JN698962 |
| *cxcl8a* | GATCTGTCTGGACCCCTCTG | GGGCATTCATGGTTTTCTGT | CCATGGGTTAAGAAGATCATTGATAGG | 79 | XM_001342570 |
| *ifng1* | TATGGGCGATCAAGGAAAAC | CTTTAGCCTGCCGTCTCTTG | CGATCGTCCAGCGAAAGGCT | 129 | AB158361 |
| *mpeg1.1* | CCCACCAAGTGAAAGAGG | GTGTTTGATTGTTTTCAATGG | TCTGGACTCCCGCTTTGCAA | 150 | NM_212737 |
| *mpeg1.2* | ATGGAGAGTGGCAAACCTTG | TTTTCCCCTCCTGACATCTG | TGGCCAAACTGATCGATGCA | 90 | NM_001020586 |
| *rpl13a* | TCCCAGCTGCTCTCAAGATT | ACTTCCAGCCAACTTCATGG | CACACGCAAATTTGCCCTGC | 87 | NM_212784 |
| *saa* | CTTGCTGTGCTGGTGATGTT | CTTCCAATTGGCCTCTTTCA | CGCTGGAGGTGCAAAGGACA | 129 | BC081487 |
| *tgfb1a* | TGCGCAAGCTTTACATTGAC | AGGACCCCATGCAGTAGTTG | TGGATCCACAAGCCCAAGGG | 93 | AY178450 |
| *tlr2* | TGTCTCCCACCCTGAAACTC | GCCACTCTCCTATCCCAACA | CCTCCACGACCGATCAAGCC | 227 | AY388399 |
| *tlr3* | AAAGGGCTACGTTTGGTGTG | GTTGGTGGAGTTCAGCCATT | TCGCAAGAATTTTCGCCATTTTG | 119 | BC107955 |
| *tnfa* | GCGCTTTTCTGAATCCTACG | AAGTGCTGTGGTCGTGTCTG | TGCACGCAGGAGCCTGAATC | 169 | AY427649 |
