# Supplementary material for: Association between adaptive immunity and neutrophil dynamics in zebrafish (Danio rerio) infected by a parasitic ciliate
Source: PLoS One. 2018 Sep 11;13(9):e0203297. doi: 10.1371/journal.pone.0203297 (PMC6133357; doi:10.1371/journal.pone.0203297)

**Association between adaptive immunity and neutrophil dynamics in zebrafish (*Danio rerio*) infected by a parasitic ciliate**

Louise von Gersdorff Jørgensen, Rozalia Korbut, Sandra Jeberg, Per Walter Kania & Kurt Buchmann

S1 Fig


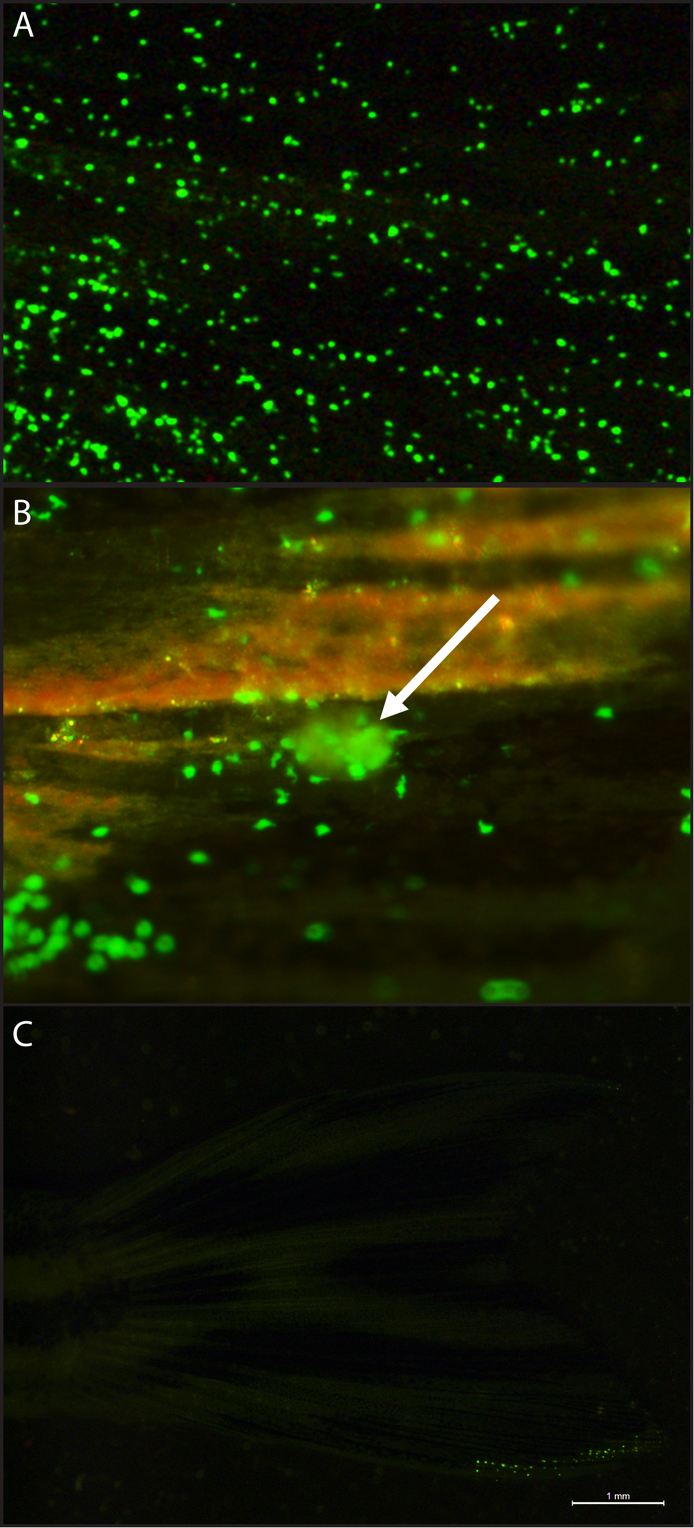

Supplement: S1 Fig — A) Enlargement of a section on a fin from Fig 5 (0h, IC) to show the neutrophils in the transgenic zebrafish line Tg(MPO:GFP)i114 in greater detail. B) Neutrophil accumulation in connection with I. multifiliis on the tail fin of an adult zebrafish of the same transgenic line. C) A wild type zebrafish tail fin using GFP filter illumination. The white arrow indicates the parasite. (DOCX) [file pone.0203297.s003.docx]
